# Supplementary material for: Long-Term Follow-Up after Mycobacterium Chimaera Infection Following Cardiac Surgery: Single-Center Experience
Source: J Clin Med. 2023 Jan 26;12(3):948. doi: 10.3390/jcm12030948 (PMC9917935; doi:10.3390/jcm12030948)
Supplement: Supplementary file 1 [file jcm-12-00948-s001.zip › jcm-2165568-supplementary.pdf]

Supplementary Table S1. Detailed surgical management.

Case n°	Redo cardiac surgery	Substitute	CPB arterial cannulation site	CPB Time (min)	Aortic cross-clamp time (min)	Circulatory arrest time (min)	Extracorporeal cytokine adsorber	Second redo cardiac surgery	Substitute
**1**	Aortic root replacement<br>Permanent epicardial PM implantation	Aortic homograft	Aorta	138	118	*NA*	No	*NA*	
**2**	Aortic root replacement	Aortic homograft	Aorta	160	141	*NA*	No	*NA*	
**3**	Annuloplasty ring and Gore –Tex neo-chordae extraction<br>Mitral valve reconstruction	Annuloplasty ring<br>Gore-Tex neo-chorda	Femoral artery	323	200	*NA*	Yes	Annuloplasty ring extraction<br>Mitral chordae transfer	Autologous
**4**	Aortic root, AA, and hemiarch replacement<br>Permanent PM extraction, epicardial PM implantation	Aortic homograft<br>Bovine pericardial tube	Aorta	367	272	22	Yes	*NA*	
**5**	Aortic root, AA, and hemiarch replacement	Aortic homograft<br>Bovine pericardial tube	Aorta	161	137	10	Yes	*NA*	
**6**	Aortic root and AA replacement	Aortic homograft	Aorta	193	164	*NA*	Yes	*NA*	
**7**	AA and hemiarch replacement	Aortic homograft	Right axillary artery	133	66	14	Yes	Distal AA replacement	Bovine pericardial tube

PM: pacemaker
AA: ascending aorta
CPB: cardiopulmonary bypass
NA: not applicable
